# Synthesis and Antibacterial Activity of 3-(Substituted)-2-(4-oxo-2-phenylquinazolin-3(4H)-ylamino)quinazolin-4(3H)-one

**DOI:** 10.1155/2016/1249201

**Published:** 2016-04-07

**Authors:** Ramgopal Appani, Baburao Bhukya, Kiran Gangarapu

**Affiliations:** ^1^Department of Pharmaceutical Chemistry and Phytochemistry, Nethaji Institute of Pharmaceutical Sciences, Somidi, Kazipet, Warangal, Telangana 506003, India; ^2^Department of Pharmaceutical Chemistry, Chaitanya Institute of Pharmaceutical Sciences, Rampur, Warangal, Telangana 506151, India

## Abstract

A series of novel 3-(substituted)-2-(substituted quinazolinylamino)quinazolin-4(3H)-ones were synthesized by the reaction of 3-(substituted)-2-hydrazino-quinazoline-4(3H)-ones with 2-phenyl-3,1-benzoxazin-4-one. The starting materials 3-(substituted)-2-hydrazino-quinazolin-4(3H)-ones were synthesized from various primary amines by a multistep synthesis. All the title compounds were tested for their antibacterial activity using ciprofloxacin as reference standard. Compounds 3-(4-fluorophenyl)-2-(4-oxo-2-phenylquinazolin-3(4H)-ylamino)quinazolin-4(3H)-one (**9a**) and 3-(4-chlorophenyl)-2-(4-oxo-2-phenylquinazolin-3(4H)-ylamino)quinazolin-4(3H)-one (**9h**) emerged as the most active compounds of the series. These compounds have shown most potent antibacterial activity against the tested organisms of* Proteus vulgaris* and* Bacillus subtilis* having zone of inhibition values of 1.1 cm and 1.4 cm for compound** 9a** 1.2 cm and 1.0 cm for compound** 9h**, respectively.

## 1. Introduction

Quinazolinones are an interesting class of heterocyclic compounds that have great importance in medicinal chemistry and reported to possess a diverse range of pharmacological activities such as antimicrobial [1–5], anti-inflammatory [6], antioxidant [7], anticonvulsant [8], antihypertensive [9], bronchodilator [10], and anticancer activities [11]. The quinazoline-4-one moiety has shown versatile pharmacodynamic action in many of its synthetic analogues as well as in many isolated derivatives of naturally occurring alkaloids [12].

Quinazolinones are one of the fused heterocyclic compounds that are of considerable interest because of diverse range of biological activities [13, 14]. This fused bicyclic compound was earlier known as benzo-1,3-diazine [15]. Imines (Schiff bases) and their reaction products are an important class of the most widely used organic compounds and have been studied over the years and reported to possess a wide range of biological activities such as anti-inflammatory [16], anticonvulsant [17], antimicrobial [18], antioxidant [19], cytotoxic [20], and antitubercular [21].

## 2. Materials and Methods

Reactions were monitored by thin-layer chromatography (TLC) on precoated silica gel GF254 plates from E-Merck Co. and compounds visualized either by exposure to UV or by dipping in 10% aqueous potassium permanganate solution. Melting points were determined using open capillary tube method and are uncorrected. Infrared (IR) spectra were recorded using KBr disk on a Thermo Nicolet MX-1 FTIR spectrometer. ^1^H NMR spectra were recorded on Bruker AMX-400 and 101 MHz, respectively. Chemical shifts are reported in *δ* ppm units with respect to TMS as internal standard. Mass spectra were recorded on Autospec Mass Spectrometer under the electron impact at 70 eV. All the chemicals used were of analytical grade obtained from Himedia and SD fine chemicals.

**Scheme 1:**
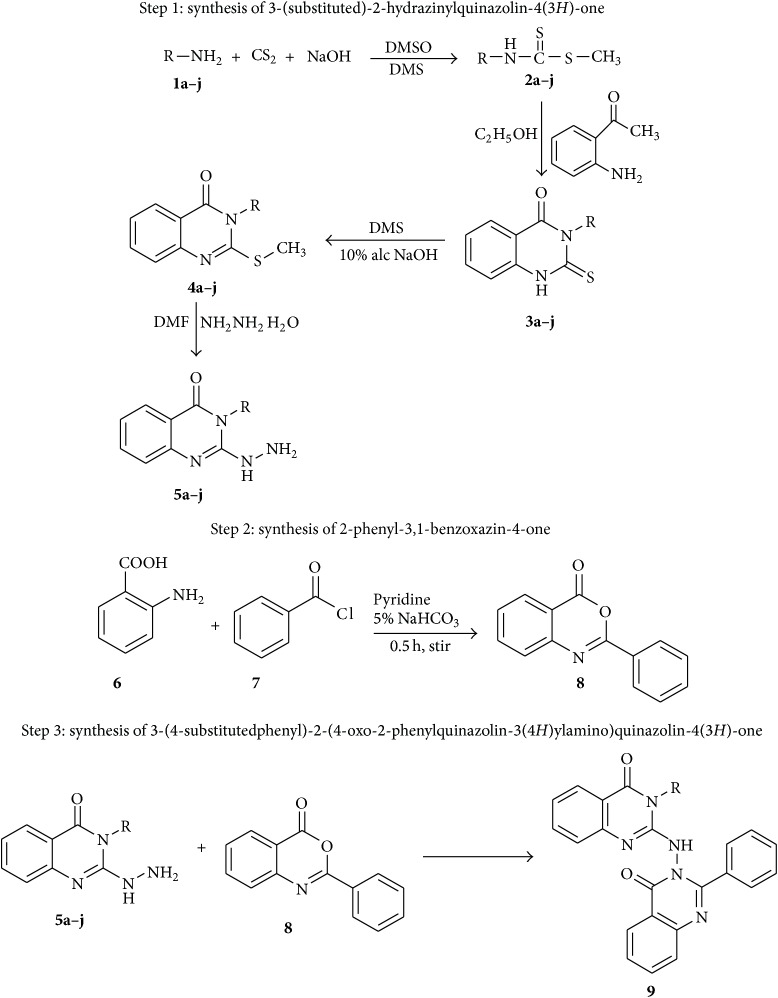


The title compounds were synthesized by the synthetic route depicted in Scheme 1.

### 2.1. Experimental

*General Procedure*

*Synthesis of 3-(4-Substituted phenyl)-2-thioxoquinazolin-4(3H)-one (* ***3a***–***3j***). A solution of substituted aniline (**1a**–**1j**) (0.02 mol) in dimethyl sulfoxide (10 mL) was stirred vigorously. To this simultaneously carbon disulphide (1.6 mL) and aqueous sodium hydroxide 1.2 mL (20 molar solutions) were added dropwise during 30 min with stirring. Dimethyl sulphate (0.02 mol) was added gradually by keeping the reaction mixture stirring in a freezing mixture for 2 h. The reaction mixture was then poured into ice water. The solid obtained was filtered, washed with water, dried, and recrystallized from ethanol. The above prepared dithiocarbamic acid (**2a**–**2j**) (0.01 mol) was dissolved in ethanol; to this methyl anthranilate (0.02 mol) was added and refluxed for 16 h; completion of the reaction was ascertained by the ceasing of methyl mercaptan evolution. The reaction mixture was cooled to room temperature; the solid (**3a**–**3j**) obtained was filtered, washed with water, dried, and recrystallized from ethanol.

*Synthesis of 3-(4-Substituted phenyl)-2-(methylthio)quinazolin-4(3H)-one (* ***4a***–***4j***). The compound (**3a**–**3j**) (2.7 g, 0.02 mol) was dissolved in alcoholic NaOH solution with continuous stirring. Dimethyl sulphate (DMS) (0.02 mol) was added drop by drop to the stirring solution for 10 min and was allowed to stir vigorously for 3 h. The solution was then added to crushed ice and left undisturbed in the refrigerator overnight. The solution was filtered on the next day and the product (**4a**–**4j**) thus obtained was dried.

Mo F: C_15_H_11_FN_2_OS, Mol Wt: 286, M.P: 153–155°C, % Yield: 81%, IR (KBr) cm^−1^: 1682 (C=O), 1603 (C=C) cm^−1^, ^1^H NMR (CDCl_3_) *δ*: 2.11 (s, 3H, SCH_3_), 6.92–8.35 (m, 8H, ArH) and MS (*m*/*z*): 286 (M^+^).

*Synthesis of 3-(4-Fluorophenyl)-2-hydrazinylquinazolin-4(3H)-one (* ***5a***–***5j***). The compound (**4a**–**4j**) (0.01 mol) was dissolved in dimethyl formamide (DMF) and to it hydrazine hydrate (0.01 mol) was added; potassium carbonate (100 mg) was added as catalyst and was refluxed on the mantle for 45 h (until disappearance of methyl mercaptan evolution). After complete evolution of methyl mercaptan the solution was cooled and filtered and the product (**5a**–**5j**) was dried.

Mol F: C_14_H_11_N_4_OF, Mol Wt: 270, *R*_*f*_ Value: 0.39 (Benzene : Chloroform : Methanol) (2 : 1 : 0.3), Melting Point: 174–176°C, % Yield: 72%, IR (KBr) cm^−1^: 3310, 3226 (NHNH_2_), 1680 (C=O) cm^−1^, ^1^H NMR (CDCl_3_) *δ*: 4.50 (s, 2H, NH_2_), 7.32–8.35 (m, 8H, ArH), 8.62 (s, 1H, NH) and MS (*m*/*z*): 270 (M^+^).

*Synthesis of 2-Phenyl-3,1-benzoxazin-4-one (* ***8***). To the solution of anthranilic acid (0.1 mol) dissolved in pyridine (60 mL), benzoyl chloride (0.2 mol) was added. The mixture was stirred for 0.5 h followed by treatment with 5% NaHCO_3_. The separated solid (**8**) was recrystallized from ethanol.

Mol F: C_14_H_9_NO_2_, Mol Wt: 223, *R*_*f*_ Value: 0.89 (Benzene : Chloroform : Methanol) (2 : 1 : 0.3), M.P: 103–105°C and % Yield: 82%.

*Synthesis of 3-(4-Substituted phenyl)-2-(4-oxo-2-phenylquinazolin-3(4H)-ylamino)quinazolin-4(3H)-one (* ***9a***–***9j***). The compound (**5a**–**5j**) and compound (**8**) were taken in equimolar amount and dissolved in glacial acetic acid and potassium carbonate anhydrous (100 mg) was added as catalyst and kept for reflux for about 32 h. Then the solution was added in crushed ice and filtered on the next day and the product (**9a**–**9j**) was dried.

*Spectra Data of the Synthesized Compounds*

*(1) 3-(4-Fluorophenyl)-2-(4-oxo-2-phenylquinazolin-3(4H)-ylamino)quinazolin-4(3H)-one (* ***9a***). Yield 86%; Mol F: C_28_H_18_N_5_O_2_F; Mol Wt: 475; *R*_*f*_: 0.418 (Benzene : Chloroform : Methanol) (2 : 1 : 0.3); MP: 144–146°C; IR (KBr) cm^−1^: 3267 (NH), 1691 (C=O), 1608 (C=C), 1148 (C-F); ^1^H NMR (CDCl_3_) *δ*: 6.76–8.49 (m, 17H, ArH), 9.01 (s, 1H, NH) and MS (*m*/*z*): 475 (M^+^).

*(2) 3-(4-Methoxyphenyl)-2-(4-oxo-2-phenylquinazolin-3(4H)ylamino)quinazolin-4(3H)-one (* ***9b*** *).* Yield: 75%; Mol F: C_29_H_21_N_5_O_3_, Mol Wt: 487, *R*_*f*_: 0.51 (Benzene : Chloroform : Methanol) (2 : 1 : 0.3), MP: 137–139°C, IR (KBr) cm^−1^: 3289 (NH), 2921 (CH_3_-CH), 1685 (C=O), 1612 (C=C), 1053 (C-O-C) cm^−1^. ^1^H NMR (CDCl_3_) *δ*: 2.92 (s, 3H, OCH_3_), 7.13–8.25 (m, 17H, ArH), 8.70 (s, 1H, NH) and MS (*m*/*z*): 487 (M^+^).

*(3) 3-(2-Chlorophenyl)-2-(4-oxo-2-phenylquinazolin-3(4H)-ylamino)quinazolin-4(3H)-one (* ***9c***). Yield: 88% Mol F: C_28_H_18_N_5_O_2_Cl, Mol Wt: 491, *R*_*f*_: 0.40 (Benzene : Chloroform : Methanol) (2 : 1 : 0.3), MP: 118–120°C, and IR (KBr) cm^−1 ^: 3369 (NH), 1682 (C=O), 1640 (C=C), 1016 (C-O-C) cm^−1^. ^1^H NMR (CDCl_3_) *δ*: 7.45–8.67 (m, 17H, ArH), 9.01 (s, 1H, NH) and MS (*m*/*z*): 491 (M^+^).

*(4) 3-(3-Methoxyphenyl)-2-(4-oxo-2-phenylquinazolin-3(4H)-ylamino)quinazolin-4(3H)-one (* ***9d***). Yield: 87% Mol F: C_29_H_21_N_5_O_3_, Mol Wt: 487, *R*_*f*_: 0.65 (Benzene : Chloroform : Methanol) (2 : 1 : 0.3), MP: 113–115°C, and IR (KBr) cm^−1^: 3292 (NH), 1698 (C=O), 1626 (C=C), 1001 (C-O-C) cm^−1^. ^1^H NMR (CDCl_3_) *δ*: 2.92 (s, 3H, OCH_3_), 7.15–8.48 (m, 17H, ArH), 8.82 (s, 1H, NH) and MS (*m*/*z*): 487 (M^+^).

*(5)  3-(2-Methoxyphenyl)-2-(4-oxo-2-phenylquinazolin-3(4H)-ylamino)quinazolin-4(3H)-one (* ***9e*** *).* Yield: 96%, Molecular Formula: C_29_H_21_N_5_O_3_, Molecular Weight: 487, *R*_*f*_ Value: 0.50 (Benzene : Chloroform : Methanol) (2 : 1 : 0.3), MP: 118–120°C, and IR (KBr) cm^−1^: 3364 (NH), 1690 (C=O), 1593 (C=C), 1026 (C-O-C) cm^−1^; ^1^H NMR (CDCl_3_) *δ*: 2.49 (s, 3H, OCH_3_), 7.04–8.07 (m, 17H, ArH), 8.89 (s, 1H, NH) and MS (*m*/*z*): 487 (M^+^).

*(6) 3-(4-Tolyl)-2-(4-oxo-2-phenylquinazolin-3(4H)-ylamino)quinazolin-4(3H)-one (* ***9f***). Yield: 90% Mol F: C_29_H_21_N_5_O_2_, Mol Wt: 471, *R*_*f*_: 0.50 (Benzene : Chloroform : Methanol) (2 : 1 : 0.3), Melting Point: 133–135°C, and IR (KBr) cm^−1^: 3275 (NH), 1720 (C=O), 1633 (C=C), 1016 (C-O-C) cm^−1^; ^1^H NMR (CDCl_3_) *δ*: 2.35 (s, 3H, CH_3_), 7.04–8.12 (m, 17H, ArH), 8.95 (s, 1H, NH) and MS (*m*/*z*): 471 (M^+^).

*(7) 3-(2-Tolyl)-2-(4-oxo-2-phenylquinazolin-3(4H)-ylamino)quinazolin-4(3H)-one (* ***9g***). Yield: 64% Molecular Formula: C_29_H_21_N_5_O_2_, Molecular Weight: 471, *R*_*f*_ Value: 0.46 (Benzene : Chloroform : Methanol) (2 : 1 : 0.3), MP: 114°C–117°; IR (KBr) cm^−1^: 3358 (NH), 1733 (C=O), 1541 (C=C), 998 (C-O-C) cm^−1^; ^1^H NMR (CDCl_3_) *δ*: 2.38 (s, 3H, CH_3_), 7.08–8.17 (m, 17H, ArH), 8.92 (s, 1H, NH) and MS (*m*/*z*): 471 (M^+^).

*(8) 3-(4-Chlorophenyl)-2-(4-oxo-2-phenylquinazolin-3(4H)-ylamino)quinazolin-4(3H)-one (* ***9h*** *).* Molecular Formula: C_28_H_18_N_5_O_2_Cl, Molecular Weight: 491, *R*_*f*_ Value: 0.46 (Benzene : Chloroform : Methanol) (2 : 1 : 0.3), MP: 132–134°C, % Yield: 90%, IR (KBr) cm^−1^: 3262 (NH), 1648 (C=O) cm^−1^, 1534 (C=C), 742 (C-Cl) cm^−1^, ^1^H NMR (CDCl_3_) *δ*: 7.01–8.48 (m, 17H, ArH), 9.24 (s, 1H, NH) and MS (*m*/*z*): 491 (M^+^).

*(9) 3-(Phenyl)-2-(4-oxo-2-phenylquinazolin-3(4H)-ylamino)quinazolin-4(3H)-one (* ***9i***). Yield: 90%, Mol F: C_28_H_19_N_5_O_2_, Mol Wt: 457, *R*_*f*_ Value: 0.38 (Benzene : Chloroform : Methanol) 2 : 1 : 0.3, MP: 143–145°C, IR (KBr) cm^−1^: 3242 (NH), 1698 (C=O) cm^−1^, 15341 (C=C) cm^−1^. ^1^H NMR (CDCl_3_) *δ*: 7.05–8.12 (m, 18H, ArH), 9.28 (s, 1H, NH) and MS (*m*/*z*): 491 (M^+^).

*(10) 3-(Benzyl)-2-(4-oxo-2-phenylquinazolin-3(4H)-ylamino)quinazolin-4(3H)-one (* ***9j***). Yield: 75%; Mol F: C_29_H_21_N_5_O_3_, Mol Wt: 486, *R*_*f*_ Value: 0.51 (Benzene : Chloroform : Methanol) 2 : 1 : 0.3, MP: 137–139°C, IR (KBr) cm^−1^: 3367 (NH), 1680 (C=O) cm^−1^, 1618 (C=C) cm^−1^. ^1^H NMR (CDCl_3_) *δ*: 7.28–8.74 (m, 18H, ArH), 9.20 (s, 1H, NH), 4.48 (s, 2H, -CH_2_) and MS (*m*/*z*): 486 (M^+^).

*Biological Activity*

*Antibacterial Activity.* Zone of inhibition method was determined to assess the antibacterial activity of the compound by agar cup plate method [22, 23]. Two Gram-positive* S. aureus* and* B. subtilis* and four Gram-negative* E. coli*,* P. vulgaris*,* K. pneumoniae*, and* P. aeruginosa* bacteria were used. Ciprofloxacin was used as a reference.

**Figure 1:**
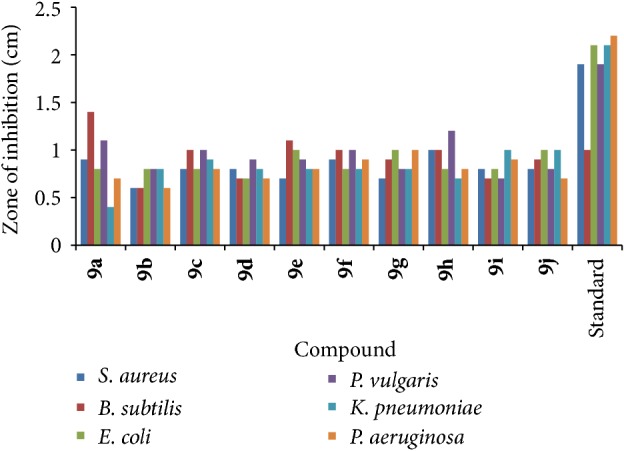
Antibacterial activity of newly synthesized compounds.

A stock solution of the newly synthesized compound (100 *μ*g/mL) in dimethyl formamide was prepared and graded quantities of the test compounds were incorporated in specified quantity of molten sterile agar (nutrient agar). A specified quantity of the medium at 40–50°C containing the compound was poured into a Petri dish to give a depth of 3-4 mm and allowed to solidify. Suspension of the microorganism was prepared to contain approximately 5 × 10^−5^ cfu/mL. The culture plates were each seeded with test organisms and allowed to solidify and thereafter punched with a sterile cork borer (5.0 mm diameter) to cut uniform wells. The open wells were filled with 0.05 mL of the test compounds and incubated at 37°C for 24 h. The zones of inhibition were then measured and recorded and compared with positive standard control ciprofloxacin. The results of the antibacterial activity containing zone of inhibition values are given in Table 1 and Figure 1.

## 3. Results and Discussion

The IR spectrum of (**3h**) shows intense peaks at 3210 cm^−1^ for cyclic thiourea (NH), 1690 cm^−1^ for carbonyl (C=O), and 1210 cm^−1^ for thioxo (C=S) stretching. ^1^HNMR spectrum of (**3h**) showed for aromatic (8H) protons a multiplet at *δ* 7.5–8.2 ppm and a singlet at *δ* 10.36 ppm indicating the presence of NH. The IR spectrum of (**4h**) showed disappearance of NH and C=S stretching signals of the compound (**3h**). It showed a peak of carbonyl (C=O) stretching at 1680 cm^−1^. The ^1^H-NMR spectrum of compound (**4h**) showed singlet at 2.7 *δ* ppm due to methyl group and multiplet at 7.3–8.4 *δ* ppm due to aromatic protons. The IR spectrum of (**5h**) showed disappearance of C-S signals of the starting material. It showed a peak of carbonyl (C=O) stretching at 1680 cm^−1^. The ^1^HNMR spectrum of compound (**5h**) showed singlet at 4.98 *δ* ppm amino group, multiplet at 6.93–8.12 *δ* ppm due to aromatic protons, and singlet at 8.73 *δ* ppm due to secondary amino group.

The title compounds (**9a**–**9j**) were obtained in fair and good yields through the condensation compound (**8**) with a variety of substituted hydrazines (**5a**–**5j**) using glacial acetic acid as a solvent.

The formation of title compounds is indicated by the appearance of peaks due to secondary amino group at 3262 cm^−1^ in the IR spectra of the compounds. It also showed a peak for carbonyl (C=O) around 1648 cm^−1^. The ^1^H NMR spectra of the title compounds showed peaks for substituents at C-2 and a singlet around 9.24 *δ* ppm due to NH; a multiplet at 7.01–8.48 *δ* ppm was observed for aromatic protons.

*Antibacterial Activity.* Among the different substituents on the C-2, electron withdrawing groups exhibited better activity over the aliphatic substituents (Table 1 and Figure 1). Compounds with electron withdrawing substituents like -Cl and -F showed better activity over the unsubstituted and electron donating substituents. Compounds** 9a** and** 9h** emerged as the most active compounds of the series. Compounds** 9a** and** 9h** have shown most potent activity against* P. vulgaris *and* B. subtilis*.

## Figures and Tables

**Table 1:** Antibacterial activity (zone of inhibition) of newly synthesized 3-(substituted)-2-(4-oxo-2-phenylquinazolin-3(4H)-ylamino)quinazolin-4(3H)-ones (**9a–9j**).

Microorganisms	Test compounds (zone of inhibition 100 *µ*g/mL) in cm										Standard^*∗*^ (10 *µ*g/mL)
	**9a**	**9b**	**9c**	**9d**	**9e**	**9f**	**9g**	**9h**	**9i**	**9j**	
*S. aureus*	0.9	0.6	0.8	0.8	0.7	0.9	0.7	1	0.8	0.8	1.9
*B. subtilis*	1.4	0.6	1	0.7	1.1	1	0.9	1	0.7	0.9	1.0
*E. coli*	0.8	0.8	0.8	0.7	1	0.8	1	0.8	0.8	1	2.1
*P. vulgaris*	1.1	0.8	1	0.9	0.9	1	0.8	1.2	0.7	0.8	1.9
*K. pneumoniae*	0.4	0.8	0.9	0.8	0.8	0.8	0.8	0.7	1	1	2.1
*P. aeruginosa*	0.7	0.6	0.8	0.7	0.8	0.9	1	0.8	0.9	0.7	2.2

^*∗*^Ciprofloxacin used as a reference standard for other bacteria.
